# Antibodies Against Phosphorylcholine Among 60-Year-Olds: Clinical Role and Simulated Interactions

**DOI:** 10.3389/fcvm.2022.809007

**Published:** 2022-04-11

**Authors:** Shailesh Kumar Samal, Pritam Kumar Panda, Max Vikström, Karin Leander, Ulf de Faire, Rajeev Ahuja, Johan Frostegård

**Affiliations:** ^1^Section of Immunology and Chronic Disease, Institute of Environmental Medicine, Karolinska Institutet, Solna, Sweden; ^2^Condensed Matter Theory Group, Materials Theory Division, Department of Physics and Astronomy, Uppsala University, Uppsala, Sweden; ^3^Unit of Cardiovascular and Nutritional Epidemiology, Institute of Environmental Medicine, Karolinska Institutet, Solna, Sweden; ^4^Department of Physics, Indian Institute of Technology Ropar, Rupnagar, India

**Keywords:** antibodies, phosphorylcholine (PC), stroke, myocardial infarction, immune system, molecular docking and dynamics, SAbPred

## Abstract

**Aims:**

Antibodies against phosphorylcholine (anti-PC) are implicated as protection markers in atherosclerosis, cardiovascular disease (CVD), and other chronic inflammatory conditions. Mostly, these studies have been focused on IgM. In this study, we determined IgG, IgG1, and IgG2 anti-PC among 60-year-olds.

**Methods:**

Based on a 7-year follow-up of 60-year-olds (2,039 men and 2,193 women) from Stockholm County, we performed a nested case-control study of 209 incident CVD cases with 620 age- and sex-matched controls. Anti-PC was determined using ELISA. We predicted the binding affinity of PC with our fully human, in-house-produced IgG1 anti-PC clones (i.e., A01, D05, and E01) using the molecular docking and molecular dynamics simulation approach, to retrieve information regarding binding properties to PC.

**Results:**

After adjustment for confounders, IgG and IgG2 anti-PC showed some significant associations, but IgG1 anti-PC was much stronger as a protection marker. IgG1 anti-PC was associated with an increased risk of CVD below 33rd, 25th, and 10th percentile and of stroke below 33rd and 25th, and of myocardial infarction (MI) below 10th percentile. Among men, a strong association with stroke was determined below the 33rd percentile [HR 9.20, CI (2.22–38.12); *p* = 0.0022]. D05 clone has higher binding affinity followed by E01 and A01 using molecular docking and further have been confirmed during the course of 100 ns simulation. The stability of the D05 clone with PC was substantially higher.

**Conclusion:**

IgG1 anti-PC was a stronger protection marker than IgG anti-PC and IgG2 anti-PC and also separately for men. The molecular modeling approach helps in identifying the intrinsic properties of anti-PC clones and atomistic interactions with PC.

## Introduction

**FIGURE 1:**
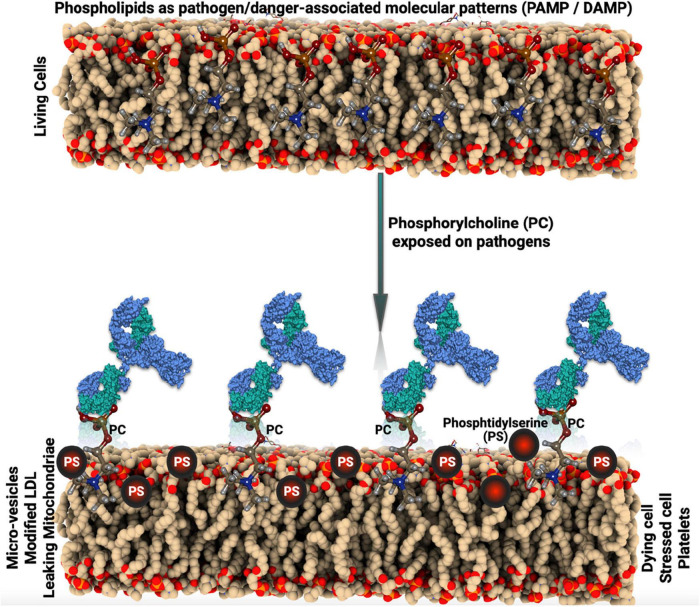
Schematic illustration of the pathogen-associated molecular pattern (PAMP) and danger-associated molecular pattern (DAMP) mechanism.

Phosphorylcholine (PC) is a danger-associated molecular pattern (DAMP), exposed on oxidized phospholipids as in oxidized low-density lipoprotein (OxLDL) and on apoptotic cells (1). In addition, PC is a pathogen-associated molecular pattern (PAMP) and an important antigen on bacteria, for example, *Streptococcus pneumoniae* and parasites and nematodes (Figure 1). PC is only recognized by the immune system and antibodies when exposed, bound to carriers, which can be lipids, proteins, or carbohydrates (1). IgG and IgM antibodies against PC (anti-PC) are present in healthy adults, at relatively high levels (1).

We have reported that IgM anti-PC is a protection marker for several chronic inflammatory disease conditions, including atherosclerosis and cardiovascular disease (CVD), rheumatic diseases, especially systemic lupus erythematosus (SLE), and mortality in chronic kidney disease (1–5). These findings have been largely confirmed by other groups (6–9). We recently reported that IgG1, but not IgG2, has similar associations with protection as IgM, in atherosclerosis (10), SLE (11), and chronic kidney disease (CKD) (12). Several potential underlying mechanisms have been reported (4, 5, 10, 11, 13–15).

We recently investigated the human anti-PC repertoire and generated fully human monoclonal anti-PC. In contrast to previous reports on laboratory mice, humans had somatically mutated anti-PC using a wide variety of Ig genes (16). We reported that these clones vary in binding capacity to PC and, in some cases, promote phagocytosis of dead cells (11).

To elucidate the mechanism of affinity and binding of PC to anti-PC clones, molecular modeling approaches, e.g., molecular docking and molecular dynamics simulations studies, help understand the functional profile of individual anti-PCs and their intrinsic atomistic interactions with PC. We thus investigated information about metabolic pathways, crystal structures, binding to proteins and other compounds, and relationships of drug targets by the use of these methods (17, 18). Through phenotypic studies of text mining and chemical structure, links between different compounds can be determined. In this study, we reported that anti-PC, especially IgG1 anti-PC, is a protection marker for CVD among 60-year-olds and determined the interaction of IgG1 anti-PC clones with PC using bioinformatics approaches.

## Materials and Methods

### Subjects

The 60-year-old cohort is a large group study of cardiovascular outcomes as described (5). In 1997/1998, every third individual living in Stockholm County at the age of 60 was asked to participate in a screening accessing their cardiovascular health. A total of 4,228 participants (2,036 men and 2,192 women) were investigated for anthropometric, metabolic, and lifestyle factors. Serum and blood samples were collected (stored at –80°C). Until 2005, 211 new incidences of CVD were recorded in this cohort by matching with national registries. These include fatal and non-fatal myocardial infarction (MI), stroke, and angina pectoris. For each case, three healthy controls matched for age, sex, and other risk factors were selected randomly for a nested case-control design (5). The study was approved by the Karolinska Institutet research ethics committee and is in accordance with the Declaration of Helsinki. All subjects gave informed consent before entering the study.

To record incident cases of first CVD, new events of coronary heart disease, including fatal and non-fatal MI and ischemic stroke and hospitalization for angina pectoris, were registered. The study based on 4,232 subjects was matched with the national cause-of-death registry (fatal events until December 31, 2003) and the national in-hospital registry (non-fatal events until December 31, 2005). Through these matching procedures, 211 incident cases of CVD were recorded. Only living subjects without a history of CVD prior to recruitment were included in the matching procedures. The International Classification of Diseases (ICD-10) was used to register coronary heart disease deaths (i.e., I 20, I 21, and I 46), MI (i.e., I 21), angina pectoris including percutaneous coronary interventions and coronary artery bypass grafts (i.e., I 20, Z 95.5, and Z 95.1), and ischemic stroke (i.e., I 63–I 66). For each case, 3 controls were randomly selected, matched for sex and age (60 days). Thus, a nested case-control design (with 211 cases and 633 controls) was applied for the epidemiological and statistical analyses, and 209 cases and 620 controls were available for testing of the IgG, IgG1, and IgG2 anti-PC levels.

### Antibody Determination

IgG, IgG1, and IgG2 anti-PC were determined using ELISA essentially as described previously (11). Pooled serum from Sigma Aldrich (St Louis, MO, United States) was used as a standard control for each plate. Another sample from a control group was used as an internal control for each plate. The ratio of internal control and standard control was used to determine the coefficient of variation (CV) between the plates. The CV between the plates was kept below 10%. The concentration of the antigen used in each well was 10 μg/ml. Nunc Immuno microwell plates (Thermo Labsystems, Franklin Lakes, MA, United States) were coated with PC-bovine serum albumin (BSA). Coated plates were incubated overnight at 4°C. After washing four times with wash buffer [1 × phosphate-buffered saline with Tween^®^ 20 (PBST)], the plates were blocked with 2% BSA-phosphate-buffered saline (PBS) for 1 h at room temperature. We followed the same washing steps, and then serum samples were diluted for IgG, IgG1, and IgG2 (1:200, 1:100, and 1:100, respectively) in 0.2% BSA-PBS and added at 100 μl/well. Plates were incubated at room temperature for 2 h and washed as described above. Biotin-conjugated mouse anti-human IgG, mouse anti-human IgG1, mouse anti-human, IgG2 (diluted 1:25,000, 1:800, 1:25,000, respectively, in 1% BSA-PBS) were added at 100 μl/well and incubated at room temperature for 2 h. After four washings, the plates were incubated with incubated with horseradish peroxidase-conjugated streptavidin (1:6,000, 1:3,000, and 1:5,000, respectively, in 0.2% BSA-PBS) (Thermo Fisher Scientific, Roskilde, Denmark) at 100 μl/well for 20 min. The color was developed by adding the horseradish peroxidase substrate, 3,3′,5,5′-tetramethylbenzidine (TMB) (3.30, 5.50; Sigma Aldrich) at 100 μl/well and incubating the plates for 10, 10, and 15 min, respectively, at room temperature in the dark. The further reaction was stopped with stop solution 1 N H_2_SO_4_ at 50 μl/well. Finally, plates were read on an ELISA Multiscan Plus spectrophotometer (Spectra Max 250; Molecular Devices, San Jose, CA, United States) at 450 and 540 nm for IgG, and IgG1, IgG2, with the Biotek 800 TS absorbance reader at 450 and 630 nm. The delta value was determined *via* the subtraction of the blanked OD at 630 nm from the blanked optical density (OD) at 450 nm. The delta value of the respective sample was then divided by the delta value of the standard to reach a relative unit value of the abundance of antibodies in this sample. All samples were measured in duplicate within a single assay, and the CV between the duplicates was below 15% for all the antibodies.

### In House Generated IgG1 Anti-phosphorylcholine Clones

We used our monoclonal antibodies (mAbs), which we produced, as described (16). As in our recent publication, we used three mAbs, namely, A01, D05, and E01, which were isolated from single PC-reactive B cells from healthy human donors and which differed in binding properties to PC (11). In brief, we synthesized and cloned the cDNAs expression vectors containing human Igγ, Igλ, or Igκ. Antibodies were then produced by co-transfection of exponentially growing human embryonic kidney (HEK) cells, and then the proteins were purified using the Protein G chromatography column. Antibody protein purity and the expression of heavy (H) and light (L) chains were then confirmed by sodium dodecyl sulfate-polyacrylamide gel electrophoresis (SDS-PAGE), and affinity to PC was measured by surface plasmon resonance on Biacore X-100 (GE Healthcare, Uppsala, Sweden) with isotype control as described (16).

### Computational Methods

We have performed IgBLAST against the heavy (H) and light (L) chain sequences, e.g., E01, A01, and D05 clones derived from healthy subjects. The sequences of the clones were described in our previous work (11, 16). The hits were aligned based on the International ImMunoGeneTics information system human (IMGT), V, D, and J genes (F + ORF). The protein sequences of the H chain and L chains derived from the alignments were subjected to the antibody structure prediction using the SAbPred tool (19). Antibody informatics tools, e.g., SAbPred, helps in improving our understanding of immune responses to disease and aid in the design and engineering of therapeutic molecules. Furthermore, we have retrieved the structure of the PC molecule from PubChem database (20) and refined using Marvin Sketch for the molecular docking analysis. The docking has been performed using the AutoDock Vina tool (21) where the intrinsic atomistic interactions and binding orientations of PC with antibodies have been deciphered. Furthermore, to validate the binding conformation of PC with the clone antibody structure, we have selected the best binding conformation of PC (antigen) with a high binding affinity score and subjected it to molecular dynamics simulation using Gromacs version 2020 (22). We obtained topologies from the CGenFF database (23) for the PC molecule. The CHARMM36 force field has been used to optimize the parameters of the target receptors (i.e., E01, A01, and D05 anti-PC clones). The complex systems have been mounted on a standard, solvent-molecular cubic frame. The Ewald (PME) particle mesh method was employed to compensate for the long range of electrostatic interactions under periodic boundary conditions with the 15 Å cutoff for non-bonded contacts. A total of 0.05 ns were simulated with a time step of 1 fs. Neighbor searching was performed every 1 step. The PME algorithm was used for electrostatic interactions with a cutoff value of 1.2 nm. A reciprocal grid of 72 × 72 × 72 cells was used with fourth-order B-spline interpolation. A single cutoff value of 1.2 nm was used for Van der Waals interactions. To achieve equilibrium, the system has been neutralized with Na^+^ and Cl^–^ ions equivalents. Energy minimization and equilibration were performed by three steps as follows: (i) the entire system of ions, solvents, antibody, and PC was minimized for up to 50,000 steps using a steep algorithm, (ii) constraints were added to the antibody, and the conjugate for 100 ps during heating using a number of atoms, volume, and temperature (NVT), and (iii) at constant pressure (1 bar) and temperature (300 K) for 100 ps, with a time step of 2 fs in the equilibrium phase, a number of atoms, pressure, or temperature (NPT) ensemble were used. The algorithm SHAKE was used to restrict hydrogen to heavy atomic contacts. A total of 100 ns were simulated with a time step of 2 fs. Neighbor searching was performed every 20 steps. The PME algorithm was used for electrostatic interactions with a cutoff value of 1.2 nm. A single cutoff value of 1.223 nm was used for Van der Waals interactions. Temperature coupling was carried out with the V-rescale algorithm. Pressure coupling was carried out with the Parrinello-Rahman algorithm. In addition, the protein-linking energy was evaluated after 100 ns simulation to measure non-bonding energy interaction and short-range on-associated energy that was quantifiably reproduced in Gromacs energy profiles. Furthermore, the BSA conjugated with PC bearing PDB ID: 2BIB has been taken to study the antibody-antigen interaction to understand the mechanism of PC binding to clone antibodies in the presence of BSA. The antibody-antigen interactions were carried out using the Cluspro2.0 tool (24). The Cluspro 2.0 tool rotates the ligand with 70,000 rotations. For each rotation, it translates the ligand in x, y, and z relative to the receptor on a grid. Then, it chooses the translation with the best score from each rotation. Of the 70,000 rotations, the 1,000 rotation/translation combinations that have the lowest score were chosen. The algorithm is based on the greedy clustering of these 1,000 ligand positions with a 9-angstrom C-alpha root mean square deviation (RMSD) radius. The energy is based on repulsive, attractive, electrostatics, and Decoys as the Reference State (DARS) forces combined to give a negative score with high binding affinity. The more the negative score, the better the binding and involvement of cluster members.

### Statistical Analysis

Various data analyses, including demographic biochemistry and anthropometry related, were performed for cases and controls, respectively, with values expressed as mean (SD) for normally distributed parameters and medians (ranges) for parameters that were not normally distributed after logarithmic transformation. Statistical differences between cases and controls were evaluated through parametric tests. Odds ratios (OR) with 95% CI were calculated applying conditional logistic regression with anti-PC levels divided into percentiles as indicated. For the analyses of specific percentiles, the remaining values formed the reference. Analyses were run crude or adjusted for traditional risk factors as indicated. These analyses were performed using the SAS 9.4 release (SAS Institute, Cary, NC). For all statistical analyses, a *p* < 0.05 was considered significant.

## Results

### Clinical Associations

We identified 211 incident cases (i.e., 77 with MI, 85 with angina pectoris, and 49 with ischemic stroke) of first CVD events throughout the follow-up period. For each incident case, 3 age- and sex-matched controls were selected (i.e., 633 controls in total). Serum samples were missing for 2 cases and 13 controls, leaving 209 cases and 620 controls for analyses. As previously reported in a similar dataset, there were more hypertensives and smokers among the cases than controls and a trend-wise higher body mass index. Blood pressure level, high-density lipoprotein, and high-sensitivity C-reactive protein were associated with risk among cases as compared with those in controls. Among anti-PC determinations, only IgG1 anti-PC among men and IgG2 anti-PC among women were higher among cases than controls (*p* = 0.046 and *p* = 0.019, respectively) (Table 1). We then focused on comparisons between percentiles of anti-PC determinations.

**TABLE 1:** Baseline characteristics among incident cardiovascular disease (CVD) cases and matched controls.

	Incident cases	Controls	*P*-value
Number	209	620	NA
Age, years	60	60	NA
Male gender, %	66	66	NA
Smokers, %	32	19.7	0.0002
Diabetes %	24.4	15.6	0.005
BMI kg/m^2^	27.8 ± 4.6	26.7 ± 3.8	0.0031
Hypertension (>140/90 mm Hg), %	42.6	25.7	<0.0001
Glucose mmol/L	6.1 ± 2.5	5.6 ± 1.5	0.0004
Insulin μ mol/L	11.4 ± 7.1	10.1 ± 5.87	0.0067
Systolic blood pressure, mm Hg	148 ± 21.8	139 ± 21.2	<0.0001
Diastolic blood pressure, mm Hg	89 ± 10.6	85 ± 10.4	<0.0001
Cholesterol, mMol/l	6.1 ± 1.0	6.0 ± 1.2	0.1366
HDL, mMol/l	1.3 ± 0.4	1.4 ± 0.4	0.0005
LDL, mMol/l	4.07 ± 0.93	3.8 ± 0.94	0.04
Triglycerides, mMol/l	1.6 ± 1.0	1.4 ± 0.8	0.0005
hsCRP, mg/l	3.47 ± 10.03	2.88 ± 0.84	0.003
Anti-PC IgG unit values all	301.1 (171.1–470.2)	288.2 (161.5–458.3)	0.84
Anti-PC IgG unit values men	299.3 (160.5–454.5)	274.9 (158.7–431.9)	0.80
Anti-PC IgG unit values women	320.9 (175.8–557.1)	318 (169.5–532.5)	0.99
Anti-PC IgG1 unit values all	166.5 (97.5–283.3)	174 (112.4–282.9)	0.20
Anti-PC IgG1 unit values men	133.8 (82.6–208.6)	158 (103.7–227.4)	0.046
Anti-PC IgG 1 unit values women	264.9 (143.4–425.5)	279.5 (148.3–390.2)	0.70
Anti-PC IgG2 unit values all	203 (123.3–397.1)	213 (134.5–389.8)	0.35
Anti-PC IgG2 unit values men	255.7 (99,8–483.7)	234.7 (124.1–462.7)	0.99
Anti-PC IgG2 unit values women	159.8 (137.7–277.6)	201 (152.9–282.2)	0.019

IgG levels were divided into percentiles, and low or high levels were compared with the rest, as indicated (Table 2 and Supplementary Figure 1A). After adjustment for smoking, body mass index, type 2 diabetes mellitus, hypercholesterolemia, and hypertension, decreased risk of stroke was observed in the higher percentiles of IgG anti-PC, which was significant at the 90th percentile: OR 0.19, CI (0.04–0.98); and the *p*-value is 0.0468. We could not find significant differences in the low or high levels when compared with whole CVD events or MI/angina pectoris.

**TABLE 2:** Association between levels of IgG anti-phosphorylcholine (PC) and risk for cardiovascular disease (CVD), stroke, and myocardial infarction (MI) among all participants and men and women.

All outcomes												
Anti-PC IgG	ALL				Males				Females			
	Crude	*P*-values	Adjusted*	*P*-values	Crude	*P*-values	Adjusted*	*P*-values	Crude	*P*-values	Adjusted*	*P*-values
			
	OR (95% CI)				OR (95% CI)				OR (95% CI)			
≤10%	1.32 (0.80–2.19)	0.2744	1.47 (0.87–2.48)	0.1539	1.50 (0.83–2.69)	0.1772	**1.92 (1.03**–**3.60)**	**0.0410**	0.96 (0.36–2.56)	0.9346	0.77 (0.28–2.13)	0.6176
≤25%	0.99 (0.69–1.42)	0.9508	1.09 (0.75–1.60)	0.6441	1.00 (0.64–1.57)	0.9848	1.16 (0.72–1.87)	0.5333	0.96 (0.51–1.79)	0.8954	0.97 (0.50–1.88)	0.9270
≤33%	0.94 (0.67–1.32)	0.7097	1.00 (0.71–1.43)	0.9816	0.90 (0.60–1.37)	0.6354	1.03 (00.67–1.60)	0.8801	1.01 (0.56–1.82)	0.9800	0.91 (0.48–1.70)	0.7566
>50%	1.12 (0.82–1.53)	0.4729	1.04 (0.75–1.44)	0.8343	1.22 (0.83–1.78)	0.3083	1.06 (0.71–1.59)	0.7762	0.94 (0.55–1.63)	0.8339	1.02 (0.56–1.83)	0.9518
>66%	1.09 (0.78–1.51)	0.6306	0.92 (0.64–1.31)	0.6312	1.12 (0.74–1.69)	0.5852	0.86 (0.54–1.34)	0.4975	1.02 (0.58–1.78)	0.9432	1.05 (0.58–1.90)	0.8746
>75%	1.04 (0.71–1.52)	0.8212	0.91 (0.61–1.37)	0.6561	1.07 (0.66–1.74)	0.7730	0.83 (0.48–1.42)	0.4933	1.00 (0.55–1.83)	1.0000	1.05 (0.55–1.99)	0.8880
>90%	1.03 (0.60–1.78)	0.9067	0.80 (0.45–1.44)	0.4646	1.00 (0.47–2.13)	1.0000	0.68 (0.29–1.57)	0.3633	1.07 (0.49–2.36)	0.8653	0.96 (0.41–2.23)	0.9192

**Stroke as an outcome**												
**Anti-PC IgG**	**ALL**				**Males**				**Females**			
	**Crude**	***P*-values**	Adjusted*	***P*-values**	**Crude**	***P*-values**	Adjusted*	***P*-values**	**Crude**	***P*-values**	Adjusted*	***P*-values**
			
	**OR (95% CI)**				**OR (95% CI)**				**OR (95% CI)**			

≤10%	1.79 (0.71–4.53)	0.2197	1.80 (0.67–4.82)	0.2450	2.43 (0.69–8.64)	0.1692	2.91 (0.66–12.87)	0.1588	1.25 (0.31–5.11)	0.7556	0.86 (0.19–3.87)	0.7556
≤25%	1.09 (0.52–2.26)	0.8253	1.00 (046–2.19)	0.9981	1.20 (0.41–3.51)	0.7430	1.14 (0.35–3.74)	0.8265	1.00 (0.37–2.73)	1.0000	0.86 (0.29–2.54)	1.0000
≤33%	1.01 (0.50–2.03)	0.9763	0.98 (0.47–2.06)	0.9663	0.94 (0.35–2.51)	0.9010	1.28 (0.40–4.07)	0.6732	1.09 (0.40–2.95)	0.8651	0.80 (0.27–2.38)	0.8651
>50%	0.89 (0.46–1.71)	0.7179	0.89 (0.44–1.82)	0.7478	0.80 (0.31–2.03)	0.6355	0.52 (0.15–1.74)	0.2862	0.98 (0.39–2.46)	0.9688	1.27 (0.46–3.53)	0.9688
>66%	0.54 (0.26–1.13)	0.1034	0.47 (0.21–1.07)	0.0710	0.51 (0.17–1.53)	0.2325	0.30 (0.07–1.26)	0.0993	0.57 (0.2 1–1.53)	0.2667	00.58 (0.20–1.66)	0.2667
>75%	0.45 (0.18–1.11)	0.0825	0.38 (0.14–1.02)	0.0547	0.25 (0.05–1.26)	0.0937	**0.07 (0.01**–**0.59)**	**0.0144**	0.63 (0.21–1.90)	0.4108	0.67 (0.21–2.14)	0.4108
>90%	0.26 (0.06–1.21)	0.0855	**0.19 (0.04**–**0.98)**	**0.0468**	**N/A**	**N/A**	**N/A**	**N/A**	0.65 (0.14–3.13)	0.5926	0.54 (0.10–2.87)	0.5926

**Angina/MI as an outcome**												
**Anti-PC IgG**	**ALL**				**Males**				**Females**			
	**Crude**	***P*-values**	Adjusted*	***P*-values**	**Crude**	***P*-values**	Adjusted*	***P*-values**	**Crude**	***P*-values**	Adjusted*	***P*-values**
			
	**OR (95% CI)**				**OR (95% CI)**				**OR (95% CI)**			

≤10%	1.17 (0.64–2.14)	0.6002	1.32 (0.69–2.51)	0.3983	1.31 (0.67–2.57)	0.4220	1.67 (0.80–3.47)	0.1735	0.77 (0.20–2.99)	0.7003	0.59 (0.14–2.50)	0.4710
≤25%	0.96 (0.63–1.46)	0.8462	1.09 (0.70–1.71)	0.7019	097 (0.59–1.59)	0.9008	1.12 (0.65–1.90)	0.6887	0.93 (0.42–2.07)	0.8664	1.04 (0.44–2.45)	0.9373
≤33%	0.92 (0.62–1.35)	0.6574	0.98 (0.65–1.47)	0.9080	0.90 (0.57–1.42)	0.6420	0.98 (0.60–1.61)	0.9439	0.97 (0.47–2.0)	0.9262	0.88 (0.39–1.98)	0.7592
> 50%	1.20 (0.84–1.72)	0.3107	1.12 (0.77–1.63)	0.5567	1.33 (0.87–2.01)	0.1842	1.20 (0.77–1.86)	0.4281	0.92 (0.47–1.82)	0.8166	0.98 (0.47–2.08)	0.9665
>66%	1.32 (0.91–1.93)	0.1405	1.14 (0.76–1.71)	0.5211	1.31 (0.83–2.04)	0.2445	1.04 (0.64–1.69)	0.8821	1.38 (0.69–2.77)	0.3618	1.47 (0.69–3.13)	0.3126
> 75%	1.30 (0.85–1.99)	0.2196	1.14 (0.72–1.80)	0.5892	1.33 (0.79–2.22)	0.2818	1.05 (0.59–1.87)	0.8649	1.26 (0.60–2.64)	0.5458	1.29 (0.58–2.90)	0.5334
>90%	1.44 (0.79–2.62)	0.2385	1.08 (0.56–2.08)	0.8172	1.53 (0.70–3.35)	0.2893	1.04 (0.43–2.51)	0.9259	1.32 (0.52–3.36)	0.5653	1.08 (0.38–3.07)	0.8850
												

*Adjusted for confounders (e.g., smoking, blood pressure, and diabetes).

*Bold values mean significant.*

IgG levels on the basis of gender were compared with similar percentiles, and for men, these associations were even more pronounced. At low levels, we observed an increased risk of CVD events: below 10th percentile: OR 1.92, CI (1.03–3.60), and *p*-value is 0.0410, and in relation to stroke, a decreased risk was observed above 75th percentile: OR 0.07, CI (0.01–0.59), and *p*-value is 0.0144, while no association was observed in women.

IgG1 levels were divided into percentiles, and low or high levels were compared with the rest, as indicated (Table 3 and Supplementary Figure 1B). After adjustment for smoking, body mass index, type 2 diabetes mellitus, hypercholesterolemia, and hypertension, an increased risk of CVD was observed in the low percentiles of IgG1 anti-PC, at 10th: OR 1.80, CI (1.07–3.04), and *p*-value is 0.0272; at 25th: OR 1.62, CI (1.10–2.37), *p*-value is 0.0143, and at 33rd: OR 1.51, CI (1.05–2.15), and *p*-value is 0.0244. For stroke, the higher risk was observed at 25th: OR 2.62, CI (1.17–5.91), and *p*-value is 0.0199 and at 33rd: OR 2.97, CI (1.36–6.51), and *p*-value is 0.0065. The significant risk was observed in low levels of IgG1 anti-PC for MI/angina pectoris, the association at 10th: OR 2.20, CI (1.19–4.06), and *p*-value is 0.0116.

**TABLE 3:** Association between levels of IgG1 anti-PC and risk for CVD, stroke, and MI among all participants and men and women.

All outcomes												
Anti-PC IgG1	ALL				Males				Females			
	Crude	*P*-values	Adjusted*	*P*-values	Crude	*P*-values	Adjusted*	*P*-values	Crude	*P*-values	Adjusted*	*P*-values
			
	OR (95% CI)				OR (95% CI)				OR (95% CI)			
≤10%	**1.73 (1.06–2.84)**	**0.0291**	**1.80 (1.07**–**3.04)**	**0.0272**	**1.87 (1.07**–**3.29)**	**0.0286**	**2.05 (1.12**–**3.75)**	**0.0196**	1.33 (0.46–3.83)	0.5999	1.22 (0.40–3.68)	0.7285
≤25%	**1.51 (1.05–2.18)**	**0.0265**	**1.62 (1.10**–**2.37)**	**0.0143**	**1.53 (1.01**–**2.31)**	**0.0433**	**1.72 (1.11**–**2.67)**	**0.0159**	1.44 (0.66–3.14)	0.3556	1.33 (0.59–2.99)	0.4886
≤33%	**1.49 (1.06–2.09)**	**0.0209**	**1.51 (1.05**–**2.15)**	**0.0244**	**1.55 (1.05**–**2.28)**	**0.0287**	**1.69 (1.11**–**2.56)**	**0.0140**	1.33 (0.67–2.66)	0.4165	1.08 (0.52–2.25)	0.8356
>50%	0.87 (0.63–1.21)	0.3989	0.80 (0.57–1.13)	0.2008	0.81 (0.54–1.20)	0.2901	0.70 (0.45–1.07)	0.0960	1.02 (0.57–1.85)	0.9398	1.07 (0.57–1.98)	0.8413
>66%	0.92 (0.65–1.32)	0.6605	0.87 (0.60–1.27)	0.4747	0.80 (0.50–1.29)	0.3637	0.69 (0.42–1.13)	0.1436	1.14 (0.64–2.02)	0.6616	1.27 (0.69–2.36)	0.4406
>75%	0.93 (0.63–1.38)	0.7297	0.89 (0.60–1.33)	0.5651	0.90 (0.52–1.57)	0.7127	0.79 (0.45–1.40)	0.4160	0.97 (0.56–1.68)	0.9064	1.01 (0.56–1.81)	0.9832
>90%	1.41 (0.85–2.33)	0.1791	1.39 (0.82–2.33)	0.2189	1.50 (0.64–3.51)	0.3488	1.45 (0.60–3.51)	0.4059	1.37 (0.73–2.55)	0.3251	1.36 (0.71–2.60)	0.3528

**Stroke as an outcome**												
**Anti-PC IgG1**	**ALL**				**Males**				**Females**			
	**Crude**	***P*-values**	Adjusted*	***P*-values**	**Crude**	***P*-values**	Adjusted*	***P*-values**	**Crude**	***P*-values**	Adjusted*	***P*-values**
			
	**OR (95% CI)**				**OR (95% CI)**				**OR (95% CI)**			

≤10%	1.17 (0.40–3.42)	0.7775	1.08 (0.34–3.40)	0.8967	1.88 (0.49–7.24)	0.3597	1.72 (0.35–8.44)	0.5037	0.50 (0.06–4.15)	0.5211	0.44 (0.05–4.00)	0.4634
≤25%	**2.41 (1.12–5.19)**	**0.0251**	**2.62 (1.17**–**5.91)**	**0.0199**	**2.96 (1.04**–**8.43)**	**0.0424**	**4.76 (1.26**–**17.91)**	**0.0211**	1.87 (0.60–5.85)	0.2831	1.84 (0.55–6.16)	0.3229
≤33%	**2.55 (1.23–5.28)**	**0.0117**	**2.97 (1.36**–**6.51)**	**0.0065**	**4.65 (1.62**–**13.31)**	**0.0042**	**9.20 (2.22**–**38.12)**	**0.0022**	1.21 (0.40–3.68)	0.7379	1.04 (0.31–3.53)	0.9473
>50%	0.66 (0.34–1.30)	0.2304	0.62 (0.30–1.27)	0.1882	0.46 (0.17–1.25)	0.1268	0.44 (0.14–1.37)	0.1554	0.94 (0.37–2.43)	0.9034	0.88 (0.33–2.38)	0.8057
>66%	0.70 (0.33–1.48)	0.3526	0.64 (0.29–1.41)	0.2680	0.49(0.15–1.62)	0.2432	0.29 (0.06–1.34)	0.1128	0.92 (0.34–2.48)	0.8647	1.02 (0.35–2.96)	0.9736
>75%	0.88 (0.42–1.84)	0.7315	0.81 (0.37–1.78)	0.5992	0.68 (0.21–2.28)	0.5359	0.49 (0.11–2.16)	0.3439	1.04 (0.40–2.74)	0.9344	1.14 (0.41–3.17)	0.8048
>90%	0.85 (0.30–2.42)	0.7645	0.82 (0.28–2.45)	0.7242	1.20 (0.23–6.19)	0.8275	0.95 (0.16–5.72)	0.9510	0.70 (0.18–2.66)	0.5979	0.76 (0.19–3.11)	0.7077

**Angina/MI as an outcome**												
**Anti-PC IgG1**	**ALL**				**Males**				**Females**			
	**Crude**	***P*-values**	Adjusted*	***P*-values**	**Crude**	***P*-values**	Adjusted*	***P*-values**	**Crude**	***P*-values**	Adjusted*	***P*-values**
			
	**OR (95% CI)**				**OR (95% CI)**				**OR (95% CI)**			

≤10%	**1.94 (1.11**–**3.40)**	**0.0204**	**2.20 (1.19**–**4.06)**	**0.0116**	**1.87 (1.01**–**3.47)**	**0.0469**	**2.18 (1.10**–**4.33)**	**0.0254**	2.29 (0.61–8.56)	0.2170	2.23 (0.54–9.18)	0.2651
≤25%	1.31 (0.87–1.99)	0.2007	1.45 (0.92–2.26)	0.1071	1.35 (0.86–2.12)	0.1991	1.55 (0.95–2.53)	0.0816	1.14 (0.38–3.44)	0.8111	0.93 (0.29–3.03)	0.9066
≤33%	1.27 (0.87–1.88)	0.2191	1.31 (0.87–1.98)	0.1927	1.24 (0.81–1.91)	0.3209	1.36 (0.85–2.16)	0.1970	1.41 (0.59–3.40)	0.4388	1.08 (042–2.77)	0.8718
>50%	0.95 (0.65–1.38)	0.7749	0.84 (0.56–1.25)	0.3803	0.91 (0.59–1.40)	0.6607	0.75 (0.47–1.22)	0.2450	1.08 (0.50–2.32)	0.8465	1.17 (0.52–2.66)	0.7060
>66%	1.00 (0.67–1.51)	0.9862	0.92 (0.60–1.41)	0.7076	0.89 (0.53–1.49)	0.6535	0.75 (0.43–1.28)	0.2899	1.27 (0.62–2.57)	0.5145	1.54 (0.70–3.41)	0.2870
>75%	0.96 (0.61–1.51)	0.8473	0.88 (0.55–1.42)	0.6031	0.98 (0.52–1.82)	0.9375	0.81 (0.42–1.56)	0.5232	0.93 (0.48–1.83)	0.8411	1.02 (0.49–2.12)	0.9664
>90%	1.68 (0.94–3.02)	0.0795	1.64 (0.89–3.01)	0.1145	1.64 (0,61–4.43)	0.3314	1.64 (0.57–4.73)	0.3609	1.71 (0.83–3.51)	0.1443	1.70 (0.80–3.64)	0.1694
												

*Adjusted for confounders (e.g., smoking, blood pressure, and diabetes).

*Bold values mean significant.*

IgG1 levels on the basis of genders were compared with similar percentiles, and for men, these associations were even more pronounced for CVD, stroke, and MI/angina. The stronger significant association for CVD was at 10th: OR 2.05, CI (1.12–3.75), and *p*-value is 0.0196; at 25th: OR 1.72, CI (1.11–2.67), and *p*-value is 0.0159; and at 33rd: OR 1.69, CI (1.11–2.56), and *p*-value is 0.0140. For stroke, the higher risk was observed at 25th: OR 4.76, CI (1.26–17.91), and *p*-value is 0.0211, and at 33rd: OR 9.20, CI (2.22–38.12), and *p*-value is 0.0022. For MI/angina, also higher risk was found at 10th: OR 2.18, CI (1.10–4.33), and *p*-value is 0.0254, while no association was observed in women.

IgG2 levels were divided into percentiles, and low or high levels were compared with the rest, as indicated (Table 4 and Supplementary Figure 1C). After adjustment for smoking, body mass index, type 2 diabetes mellitus, hypercholesterolemia, and hypertension, an increased risk of CVD was observed at 33rd: OR 1.47, CI (1.04–2.07), and *p*-value is 0.0285, and for stroke, the higher risk was observed in the low percentiles of IgG2 anti-PC, at 33rd: OR 2.26, CI (1.01–5.04), and *p*-value is 0.0473. There was no association for MI/angina pectoris. IgG2 levels on the basis of genders were compared with similar percentiles, and for men and women, some significant associations were seen. Among men, we observed an increased risk of stroke: at percentile 50th: OR 0.36, CI (0.14–0.91), and *p*-value is 0.0452. When divided into CVD, stroke, or MI/angina, associations did not reach statistical significance for CVD and MI/angina in men, it was interesting to see some associations were present in women for CVD below 33rd: OR 2.67, CI (1.46–4.87), and *p*-value is 0.0014; above 50th: OR 0.43, CI (0.22–0.83), and *p*-value is 0.0122, and similar for MI/angina in women below 33rd: OR 3.65, CI (1.67–8.0), and *p*-value is 0.0012; and above 50th percentile was protection with OR 0.41, CI (0.18–0.94), and *p*-value is 0.0352.

**TABLE 4:** Association between levels of IgG2 anti-PC and risk for CVD, stroke, and MI among all participants and men and women.

All outcomes												
Anti-PC IgG2	All				Males				Females			
	Crude	*P*-values	Adjusted*	*P*-values	Crude	*P*-values	Adjusted*	*P*-values	Crude	*P*-values	Adjusted*	*P*-values
			
	OR (95% CI)				OR (95% CI)				OR (95% CI)			
≤10%	1.54 (0.93–2.55)	0.0934	1.51 (0.90–2.55)	0.1196	1.60 (0.91–2.79)	0.1015	1.56 (0.87–2.80)	0.1376	1.32 (0.41–4.29)	0.6402	1.47 (0.45–4.85)	0.5268
≤25%	1.15 (0.81–1.65)	0.4411	1.23 (0.85–1.78)	0.2790	1.14 (0.75–1.74)	0.5287	1.25 (0.80–1.93)	0.3252	1.17 (0.59–2.33)	0.6549	1.19 (0.59–2.43)	0.6246
≤33%	1.34 (0.97–1.87)	0.0772	**1.47 (1.04**–**2.07)**	**0.0285**	0.99 (0.66–1.48)	0.9448	1.08 (0.70–1.66)	0.7285	**2.49 (1.40**–**4.44)**	**0.0020**	**2.67 (1.46**–**4.87)**	**0.0014**
>50%	0.81 (0.58–1.12)	0.2034	0.75 (0.53–1.06)	0.1052	1.03 (0.69–1.54)	0.8916	0.95 (0.62–1.44)	0.7978	**0.47 (0.25**–**0.87)**	**0.0169**	**0.43 (022**–**0.83)**	**0.0122**
>66%	1.04 (0.73–1.48)	0.8215	0.97 (0.67–1.42)	0.8871	1.15 (0.78–1.70)	0.4903	1.06 (0.70–1.62)	0.7754	0.70 (0.31–1.58)	0.3904	0.70 (0.30–1.61)	0.3959
>75%	1.10 (0.76–1.60)	0.5996	1.01 (0.68–1.50)	0.9659	1.18 (0.78–1.78)	0.4427	1.06 (0.68–1.64)	0.8064	0.86 (0.37–1.99)	0.7264	0.84 (0.35–2.03)	0.7041
>90%	0.95 (0.55–1.63)	0.8374	0.93 (0.53–1.64)	0.8035	1.04 (0.56–1.94)	0.8933	0.99 (0.52–1.89)	0.9763	0.70 (0.23–2.17)	0.5390	0.69 (0.21–2.34)	0.5534

**Stroke as an outcome**												
**Anti-PC IgG2**	**All**				**Males**				**Females**			
	**Crude**	***P*-values**	Adjusted*	***P*-values**	**Crude**	***P*-values**	Adjusted*	***P*-values**	**Crude**	***P*-values**	Adjusted*	***P*-values**
			
	**OR (95% CI)**				**OR (95% CI)**				**OR (95% CI)**			

≤10%	1.75 (0.63–4.89)	0.2858	1.77 (0.59–5.27)	0.3075	2.03 (0.54–7.72)	0.2979	2.05 (0.42–10.04)	0.3778	1.42 (0.28–7.07)	0.6708	1.75 (0.33–9.19)	0.5069
≤25%	1.31 (0.60–2.83)	0.5005	1.58 (0.69–3.63)	0.2794	1.67 (0.58–4.83)	0.3475	1.97 (0.56–6.97)	0.2951	1.00 (0.32–3.10)	1.0000	1.13 (0.34–3.72)	0.8449
≤33%	1.64 (0.80–3.34)	0.1752	**2.26 (1.01**–**5.04)**	**0.0473**	2.06 (0.75–5.65)	0.1587	2.93 (0.85–10.13)	0.0894	1.30 (0.48–3.55)	0.6062	1.62 (0.53–4.91)	0.3976
>50%	**0.42 (0.21**–**0.87)**	**0.0187**	**0.38 (0.18**–**0.81)**	**0.0128**	**0.36 (0.14**–**0.91)**	**0.0298**	**0.32 (0.11**–**0.98)**	**0.0452**	0.54 (0.18–1.67)	0.2864	0.43 (0.13–1.40)	0.1618
>66%	0.66 (0.31–1.40)	0.2803	0.62 (0.27–1.41)	0.2515	0.57 (0.22–1.45)	0.2338	0.52 (0.17–1.56)	0.2395	0.88 (0.26–3.03)	0.8362	0.71 (0.19–2.65)	0.6057
>75%	0.72 (0.32–1.63)	0.4269	0.69 (0.28–1.71)	0.4231	0.60 (0.22–1.62)	0.3147	0.57 (0.18–1.82)	0.3385	1.10 (0.25–4.82)	0.8990	0.95 (0.20–4.56)	0.9464
>90%	0.73 (0.23–2.37)	0.6058	0.89 (0.25–3.23)	0.8610	0.85 (0.21–3.41)	0.8181	0.98 (0.21–4.50)	0.9776	0.53 (0.06–4.90)	0.5723	0.57 (0.04–8.26)	0.6806

**Angina/MI as an outcome**												
**Anti-PC IgG2**	**All**				**Males**				**Females**			
	**Crude**	***P*-values**	Adjusted*	***P*-values**	**Crude**	***P*-values**	Adjusted*	***P*-values**	**Crude**	***P*-values**	Adjusted*	***P*-values**
			
	**OR (95% CI)**				**OR (95% CI)**				**OR (95% CI)**			

≤10%	1.48 (0.83–2.65)	0.1852	1.39 (0.75–2.58)	0.2928	1.52 (0.82–2.81)	0.1849	1.49 (0.77–2.89)	0.2383	1.23 (0.22–6.94)	0.8188	0.90 (0.15–5.66)	0.9142
≤25%	1.11 (0.74–1.66)	0.6033	1.19 (0.78–1.82)	0.4280	1.07 (0.68–1.69)	0.7702	1.17 (0.72–1.91)	0.5274	1.28 (0.54–3.04)	0.5696	1.17 (0.46–2.99)	0.7425
≤33%	1.28 (0.88–1.85)	0.1976	1.39 (0.94–2.05)	0.1027	0.86 (0.55–1.34)	0.4986	0.92 (0.57–1.49)	0.7360	**3.43 (1.67**–**7.06)**	**0.0008**	**3.65 (1.67**–**8.00)**	**0.0012**
>50%	0.97 (0.67–1.42)	0.8860	0.91 (0.61–1.35)	0.6217	1.31 (0.84–2.08)	0.2109	1.21 (0.75–1.96)	0.4302	**0.44 (0.21**–**0.93)**	**0.0310**	**0.41 (0.18**–**0.94)**	**0.0352**
>66%	1.19 (0.80–1.78)	0.3914	1.06 (0.69–1.62)	0.7935	1.35 (0.87–2.08)	0.1793	1.16 (0.73–1.86)	0.5286	0.60 (0.21–1.74)	0.3450	0.67 (0.22–1.99)	0.4655
>75%	1.25 (0.82–1.89)	0.3021	1.05 (0.67–1.64)	0.8443	1.38 (0.87–2.19)	0.1667	1.13 (0.69–1.86)	0.6329	0.77 (0.28–2.12)	0.6152	0.80 (0.28–2.31)	0.6773
>90%	1.02 (0.55–1.87)	0.9583	0.90 (0.47–1.70)	0.7369	1.10(0.55–2.21)	0.7868	0.90 (0.43–1.85)	0.7680	0.78 (0.21–2.89)	0.7147	0.82 (0.21–3.26)	0.7778
												

*Adjusted for confounders (e.g., smoking, blood pressure, and diabetes).

*Bold values mean significant.*

### Structural Modeling of Clone Antibodies

Taking into account the association results observed, we decided to proceed with in-depth analyses using our sequences and IgG1 anti-PC clones by *in silico* methods.

**FIGURE 2:**
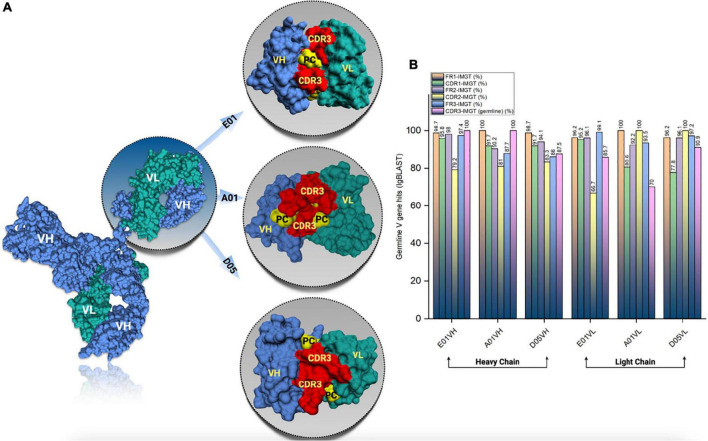
Antibody structural modeling. **(A)** The structural representation of the modelled cloned antibodies from E01, A01 and D05 clones using SAbPred. **(B)** The frequency of germline V gene hits resulted from IgBLAST analysis.

**FIGURE 3:**
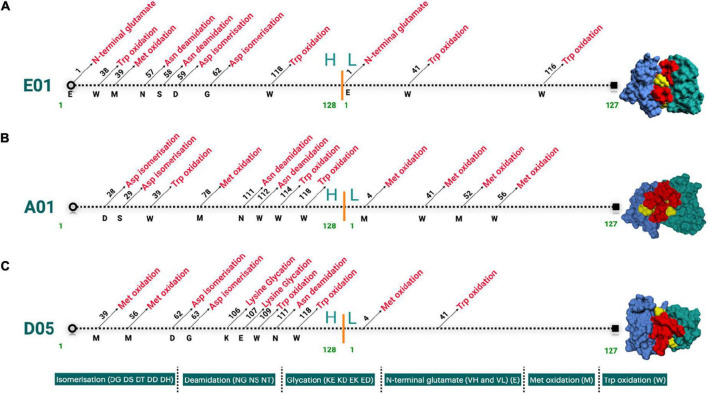
**(A–C)** Landscape of cloned antibodies sequence liabilities depicted structurally for both heavy and light chain sequences. The figure represents the sequence liabilities of various clones considered in this work, i.e., E01, A01, and D05 clones, respectively.

SAbPred analysis resulted in three structural antibody models based on VH and VL sequences from respective clones, namely, E01, A01, and D05 (Figure 2A). The resultant structural models comprise variable complementary-determining regions (CDRs), of which the CDR3 region is considered to be the most crucial part in binding specific antigens. The diversity of CDR3 amino acid sequences provides a measure of B-cell diversity in an antigen-selected B-cell repertoire. The sequence variability of the CDR3 region in all three clones was retrieved using the IgBLAST alignment. The alignment summary has been depicted in Figure 2B. From structural modeling of antibodies, we have also illustrated sequence liabilities that depict the amino acid level modifications. Among the three clones, asparagine isomerization has been observed in a high frequency. Apart from asparagine isomerization, several other modifications have been illustrated in Figures 3A–C.

**FIGURE 4:**
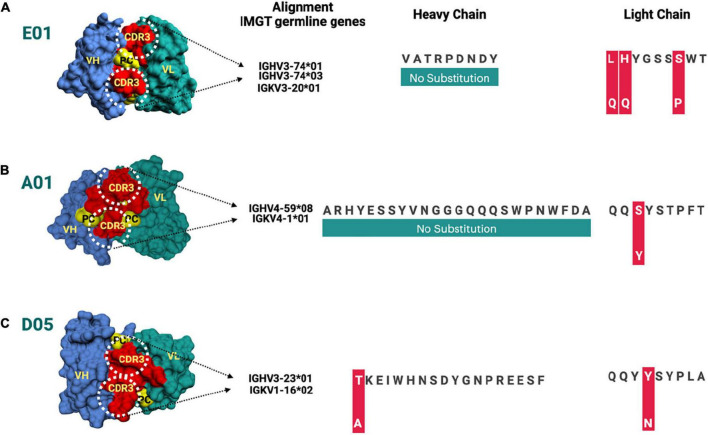
**(A–C)** Amino acid substitution map for heavy and light chain sequences compared to ImMunoGeneTics information system human (IMGT) germline genes. The amino acid substitutions are depicted for different clones, i.e., E01, A01, and D05, respectively. The red bar represents the substitution of amino acid.

One of the most prominent differences between the three clones is the lysine glycation observed only in the D05 clone, which makes it a standout. Most of the amino acid modifications were observed in H chain regions in comparison to light chain sequences. Furthermore, IgBLAST also resulted in the amino acid substitution landscape, where we observed both light and H chain sequence variability in comparison to IMGT germline genes (Figures 4A–C). D05 turns out to be different in the amino acid substitution pattern, where we have observed substitution in the H chain region. E01 and A05 do not have any substitution in the H chain region. In light chain sequence, the observed amino-acid substitution resemblance to the IMGT germline genes, i.e., IGKV3-20*01 and IGKV4-1*01, turns out to be the same in the substitution pattern, whereas D05 differs in both H and L chain cases.

### Binding Affinity of Phosphorylcholine With E01, A01, and D05 Clone Antibodies

**FIGURE 5:**
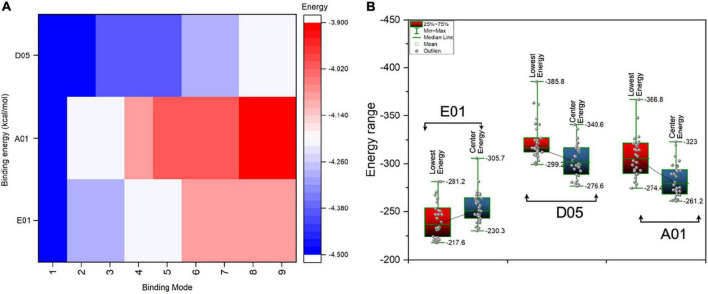
Binding energies (kcal/mol) of single molecule phosphorylcholine (PC) and PC conjugate with cloned antibodies, i.e., E01, A01, and D05. **(A)** The AutoDock Vina binding energies score of single molecule PC in kcal/mol with various clones (heatmap). The scale bar indicates the highest (indicated in blue) and lowest (indicated in red) in terms of negative score. The negative score indicated the highest binding affinity. **(B)** The box plot represents the antibody (clone antibodies)-antigen (PC conjugate) binding energies (lowest energies) for various clones.

Molecular docking of single-molecule PC with the clone antibodies reveals intrinsic atomic insights and binding orientation of PC with different CDR3 regions of clone antibodies. When the PC molecule was subjected to the molecular docking analysis with clone antibodies, the difference in binding energies has been observed (Figure 5A).

In most cases, the PC seems to interact with the CDR3 region and its vicinity. Among all the clone antibodies, the binding energies of PC with D05 clone have the highest range of binding energies, whereas the lowest have been observed in the case of the A01 clone. The nine binding modes of PC with all the clone antibodies have a range of binding energies, i.e., -3.9 to -4.5 kcal/mol. The first binding mode of PC with E01, A01, and D05 has a binding energy of -4.5 kcal/mol as the sequence variability of the clone antibodies does not differ in terms of amino acid compositions. However, when different binding modes of PC have been considered, the binding energies of PC substantially differ in all the cases. Furthermore, we have also performed the antibody-antigen interaction analysis of the PC complex conjugated with choline-binding protein E (CbpE) of *Streptococcus pneumoniae* (PDB ID: 2BIB) with the E01, A01, and D05 clone antibodies. The analysis revealed that the D05 clone has the highest binding affinity (lowest energy, i.e., -385.8 kcal/mol) toward the complex PC conjugate in comparison to other clones (Figure 5B). The interaction analyses revealed that the D05 clone has the highest binding affinity toward the single-molecule PC as well as the PC conjugate.

**FIGURE 6:**
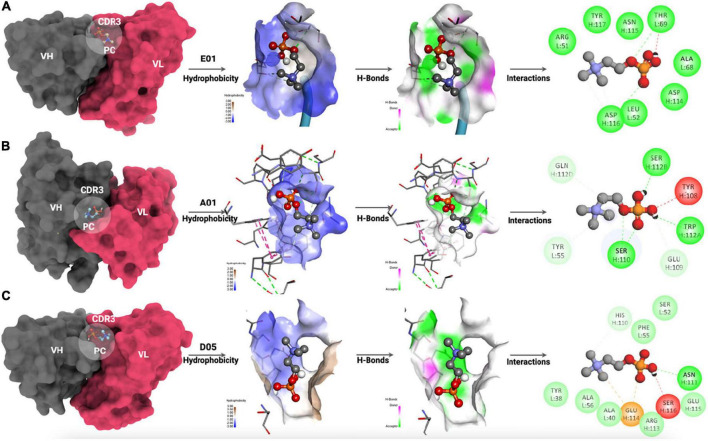
Molecular interaction of PC with **(A)** E01 clone, **(B)** A01 clone, and **(C)** D05 clone antibodies. The hydrophobicity mapping, hydrogen bonding patterns, and 2D interaction map have been illustrated.

The intrinsic atomic interaction of single-molecule PC binding to the E01, A01, and D05 clone antibodies has been illustrated in Figures 6A–C.

The interaction analyses showed the hydrophobicity mapping, hydrogen-bonding patterns, and 2D interaction map. The CDR3 region has been mapped to all the clone antibodies where PC was expected to interact. The hydrophobicity map of E01, A01, and D05 has been depicted in Figures 6A–C (right), respectively. When we compared the hydrophobicity map, the D05 clone had the highest possible hydrophobic amino acids interacting with PC as compared with E01 and A01 clones. Moreover, the hydrogen-bonding pattern of the D05 clone has a greater number of PC interacting partners among all the interactions.

### Molecule Dynamics Simulation of Phosphorylcholine With Clone Antibodies

**FIGURE 7:**
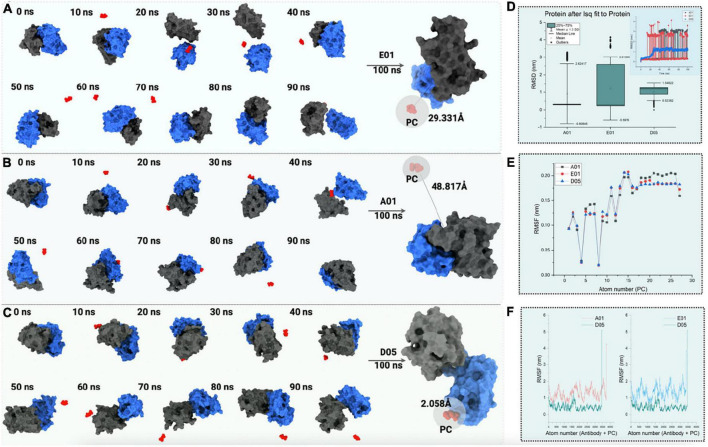
The 100 ns all-atom molecular dynamics simulation of PC with **(A)** E01, **(B)** A01, and **(C)** D05 clone antibodies. The conformations of PC binding to clone antibodies during the course of the 100 ns simulation **(D)** root mean square deviation (RMSD) analysis (nm). The box plot represents the deviation in RMSD with standard deviation depicted as mean ± 1.5 SD. **(E)** Line plot illustrating root mean square fluctuation (RMSF in nm) for PC molecule. **(F)** RMSF (in nm) for complex anti-PC clones with a comparison of D05 anti-PC clone with other clones.

With hindsight, the D05 clone stands out to be the best among all the clone antibodies in terms of molecular interactions, hydrophobicity level, and binding affinity. To further confirm the PC affinity toward the anti-PC clones, the complex has been subjected to all-atom molecular dynamics simulation using Gromacs for 100 ns (Figures 7A–C and Supplementary Video 1).

To test the binding affinity and conformation of PC, the binding mode of PC with the lowest binding energy has been considered. The root-mean-square deviation of the cloned antibody was observed to be constant throughout the simulation in the case of the D05 clone (Figure 7D). However, the solvent accessibility surface area and free energy of solvation tend to decrease as the binding affinity of PC increases. When simulated for 100 ns within the solvent environment, the PC tends to swap its position toward the CDR3 region, as shown in Figures 7A–C. As observed from the aforementioned findings, the hydrogen bonding of the PC with the D05 clone antibody increased during the 100 ns simulation followed by E01 (29.331 Å) and A01 (48.817 Å). When the hydrophobicity scale was mapped during the simulation, the final confirmation of PC tends to bind to hydrophobic amino acids as compared with the initial conformation. The analysis correlates with the 2D interaction mapping, where the PC tends to interact more with hydrophobic amino acid residues. We have also investigated the root mean square fluctuations from the perspective of the PC (Figure 7E) and the complex (Figure 7F).

In all the anti-PC clones, we found a substantial difference in terms of PC binding to the receptors. When comparing the PC binding to the anti-PC clones with respect to its stability, E01 and A01 have more deviations and thus confirm the affinity of the PC molecule toward the D05 clone.

## Discussion

We reported that low levels of anti-PC IgG1, below the 33rd percentile, are significantly associated with increased risk of CVD, including MI and stroke, where the associations for stroke were especially strong. Being a 60-year-old man and having low IgG1 anti-PC was associated with a significant and more than the ninefold increased risk of stroke within 5 years. At high levels (above 90th percentile), IgG1 anti-PC was significantly associated with protection against stroke. Associations for IgG did not reach significance, except among men, where this was only apparent at the very highest or lowest percentiles. Anti-PC IgG2 was associated with protection, among men, but also among women, reaching significance at some levels, but not as clear as IgG1 anti-PC. There were few women in this study, which was explained by the fact that the mean age for CVD is higher among women than men, about 10 years in some studies. Interestingly, anti-PC is lower among men than women in all our published studies, including among individuals from Kitava, New Guinea, living a traditional life as hunters, gatherers, and horticulturalists at the time of the investigation (1, 25–27). We have hypothesized that anti-PC, being lower in men, may contribute to the increased risk of CVD among men as compared with women at the same age (1).

As with previous studies, associations between anti-PC and various outcomes were not observed at mean levels, but rather at low levels, and in general, these findings could be interpreted as indicating that having low anti-PC levels, particularly IgM and IgG1, indicates an immune-deficient state with insufficient activity in what is typically described as natural immunity, in this case, anti-PC. We previously observed that IgM anti-PC is related to protection in this cohort (5), and the current investigation found that IgG1 anti-PC is predictive of outcome in a manner similar to IgM.

Less is known about other isotypes and subclasses of anti-PC. Our findings are in line with our previous reports, where IgG1 anti-PC were associated with protection in atherosclerosis progress (10), with the prevalence of atherosclerotic plaques, and potentially vulnerable echolucent plaques in SLE (11) and with mortality in uremia (12). We have also recently reported about the protection mechanism of anti-PC as a natural immunization against atherosclerosis in hibernating bears (28).

Animal experiments also support a protective role of anti-PC in atherosclerosis development (29), SLE (30), and RA (31). This is also supported by potential mechanisms in experimental studies, mostly performed on IgM anti-PC. These include an anti-inflammatory effect for IgG anti-PC inhibiting inflammatory lipids like platelet-activating factor (PAF) with PC as a major epitope (4), inhibition of OxLDL uptake by macrophages (5), inhibition of cell death (10), increased clearance of dead cells (13), which is also a property of anti-PC IgG1, especially, the D05 clone (11), and promotion of polarization of T regulatory cells (14). PC is playing an important role in OxLDL-induced immune activation in atherosclerosis and thus CVD (1, 10, 32). Anti-PC, especially IgM and IgG1, could thus be protective in several different chronic inflammatory diseases. We proposed development of the Hygiene/Old Friends hypothesis: lack of exposure to PC-bearing microorganisms, including nematodes, parasites, and also bacteria (including Treponema), causes low levels of anti-PC and increased risk of chronic inflammatory conditions, including atherosclerosis, CVD, and other diseases including autoimmune (1, 25–27). Low levels of anti-PC could be described as an immune-deficient state, predisposing to these conditions.

The finding in this and previous studies that IgG1 anti-PC is more associated with protection than IgG2 anti-PC is of interest and could also have implications for therapy, in addition to prediction. In general, PC can be presented as p-nitrophenyl phosphorylcholine (NPPC) (10), and human anti-PC can be divided into group I (IgM and IgG1) and group II (IgG2) (10). Interestingly, while group I anti-PC recognizes both forms of PC, group II antibodies only recognize NPCC, and the phenyl-ring attached to PC is antigenic. IgG2 anti-PC is directed against capsulated bacteria, recognizes carbohydrate antigens, and has bactericidal properties (10, 33, 34). In periodontitis, the risk of CVD is increased (35), while IgG2 anti-PC also is raised (36, 37). If our hypothesis that IgG2 anti-PC is much less of a protection marker than IgG1, this could at least partly explain why IgG2 anti-PC is mainly against PC exposed to bacterial carbohydrates.

Since anti-PC IgG1 also in this study was demonstrated to be associated with protection much more than IgG2 anti-PC, we decided to make further studies focused on this subclass, with our fully human in-house produced monoclonal anti-PC IgG1 as one basis of the studies, together with bioinformatics approaches. We recently reported that these IgG1 anti-PC clones bind differently to PC to a varying degree, increase the uptake of dead cells by efferocytosis, and inhibit proinflammatory effects of endotoxin (11, 16). In peptide analysis using a proteomics *de novo* sequencing approach, we reported differences in the CDR3 region of anti-PC IgG1 clones, which are crucial for recognition of PC on the apoptotic cell surface and other neo-epitopes (11). We currently develop these IgG1 anti-PC studies on these clones, by further analysis, with bioinformatics tools, using molecular modeling analyses of these three clones. We used the genetic sequences of their CDR region and VH and VL sequences as a basis of the analyses, which focused on structural modeling through SAbPred analysis. This resulted in three structural antibody models of CDR of which the CDR3 region is considered to be the most crucial part in binding specific antigens. The diversity of CDR3 amino acid sequences provides a measure of B-cell diversity in an antigen-selected B-cell repertoire. The sequence variability of the CDR3 region in all three clones was retrieved from IgBLAST alignment.

The diversity of CDR3 amino acid sequences provides a measure of B-cell diversity in an antigen-selected B-cell repertoire. We determined sequence liabilities and amino acid level modifications. There were several such modifications, for example, asparagine isomerization in high frequency, and there was also another modification including lysine glycation observed only in the D05 clone, which has a high-affinity binding to PC. Lysine is interesting because, in age-related disorders, glycation of macromolecules plays a vital role, especially proteins leading to their oxidation. The immunological epitopes that are impaired by the development of autoantibodies are proteins changed with glycation and glycoxidation. Protein glycation mainly leads to a stable and precocious Amadori-lysine substance to form advanced glycation end products (AGE) and is subjected to more irreversible chemical reactions (38). Most of the amino acid modifications were observed in H chain regions in comparison to light chain sequences, in the D05 clone, which thus stands out. Our previous observation that D05 is a high binder in experimental systems is in line with the observations herein.

Our findings are in line with our previous observation that in humans, anti-PC is not germline-encoded and thus in principle, not natural antibodies. In mouse models, anti-PC is germline-encoded, dominated by the one clone, T15 (39), and in line with this, knocking out this antibody is deleterious for the immune response against bacteria, which expose PC (40). Further, a mAb E06 from apolipoprotein E-knockout mouse, which is formed from OxLDL, was identical to T15 (39). We were not able to demonstrate a T15 equivalent in humans where instead anti-PC is produced by multiple B-cell subsets, with somatically mutated antibodies utilizing a wide variety of Ig-genes.

Posttranslational modifications (PTMs) of an antibody can affect an antibody’s affinity, stability, potency, and homogeneity, resulting in complicated downstream processing. The bioactivity and production of various isoforms of the product will be impacted. PTMs normally include deamidation, isomerization, oxidation, glycosylation, free thiol, pyro-glutamate, C-terminal lysine, etc. Immunogenicity, inconsistency, self-association, high viscosity, polyspecificity, or poor expression can prevent an antibody from becoming therapeutic. Early detection of these characteristics may play a pivotal role in improving the therapeutic nature of an antibody. Improved understanding of the factors regulating these biophysical properties has allowed the production of quicker *in silico* assays than their experimental counterparts (41–43).

## Limitations

The *in silico* methods used are simulations, and further experimental studies are needed to establish a clinical role of anti-PC in humans. Taken together, our findings indicate that IgG1 anti-PC is a protection marker for CVD among 60-year-olds, especially for stroke in men. We determined variations in different properties of IgG1 anti-PC clones. In the future, raising levels of anti-PC through immunization could be a promising therapeutic possibility.

## Data Availability Statement

All data needed to evaluate the conclusions in the article are present in the article and/or the Supplementary Material. The raw data supporting the conclusions of this article will be made available by the authors, without undue reservation.

## Ethics Statement

The studies involving human participants were reviewed and approved by the Karolinska Institutet research ethics committee. The patients/participants provided their written informed consent to participate in this study.

## Author Contributions

JF: conceptualization. SKS: experiments. PKP: computational analyses. MV (input from KL and UF): statistics. JF: writing (original draft preparation). SKS and PKP: co-writing. MV, KL, UF, and RA: review and editing. JF: supervision. All authors approved the submitted version of the manuscript.

## Conflict of Interest

JF was named as inventor on patents related to Phosphorylcholine. The remaining authors declare that the research was conducted in the absence of any commercial or financial relationships that could be construed as a potential conflict of interest.

## Publisher’s Note

All claims expressed in this article are solely those of the authors and do not necessarily represent those of their affiliated organizations, or those of the publisher, the editors and the reviewers. Any product that may be evaluated in this article, or claim that may be made by its manufacturer, is not guaranteed or endorsed by the publisher.
