# Supplementary material for: Antibodies Against Phosphorylcholine Among 60-Year-Olds: Clinical Role and Simulated Interactions
Source: Front Cardiovasc Med. 2022 Apr 11;9:809007. doi: 10.3389/fcvm.2022.809007 (PMC9035555; doi:10.3389/fcvm.2022.809007)
Supplement: Supplementary file 1 [file Data_Sheet_1.docx]

**Supplementary file**

**Supplemental figure 1:** Association between levels of **(A)** IgG antiPC **(B)** IgG1 antiPC **(C)** IgG2 antiPC and risk of CVD, Stroke or angina/MI, effects represented with hazard ratio (HR) and corresponding 95% confidence interval (CI). The box plot shown (left) depicts HR at 95% CI with normal distribution, optimal means and 95% CI. The heatmap (right) illustrates the association levels and HR. The color bar indicates the association level**.**
